# Li(V_0.5_Ti_0.5_)S_2_ as a 1 V lithium intercalation electrode

**DOI:** 10.1038/ncomms10898

**Published:** 2016-03-21

**Authors:** Steve J. Clark, Da Wang, A. Robert Armstrong, Peter G. Bruce

**Affiliations:** 1 Departments of Materials and Chemistry, University of Oxford, Parks Road, Oxford OX1 3PH, UK; 2 School of Chemistry and EastChem, University of St Andrews, North Haugh, Fife, Saint Andrews KY16 9ST, UK

## Abstract

Graphite, the dominant anode in rechargeable lithium batteries, operates at ∼0.1 V versus Li^+^/Li and can result in lithium plating on the graphite surface, raising safety concerns. Titanates, for example, Li_4_Ti_5_O_12_, intercalate lithium at∼1.6 V versus Li^+^/Li, avoiding problematic lithium plating at the expense of reduced cell voltage. There is interest in 1 V anodes, as this voltage is sufficiently high to avoid lithium plating while not significantly reducing cell potential. The sulfides, LiVS_2_ and LiTiS_2_, have been investigated as possible 1 V intercalation electrodes but suffer from capacity fading, large 1st cycle irreversible capacity or polarization. Here we report that the 50/50 solid solution, Li_1+*x*_(V_0.5_Ti_0.5_)S_2_, delivers a reversible capacity to store charge of 220 mAhg^−1^ (at 0.9 V), 99% of theoretical, at a rate of C/2, retaining 205 mAhg^−1^ at C-rate (92% of theoretical). Rate capability is excellent with 200 mAhg^−1^ at 3C. C-rate is discharge in 1 h. Polarization is low, 100 mV at C/2. To the best of our knowledge, the properties/performances of Li(V_0.5_Ti_0.5_)S_2_ exceed all previous 1 V electrodes.

## Abstract (web summary)

Lithium sulfides have been previously investigated as 1 V anodes for Li-ion batteries, but suffered from significant performance issues. Here, the authors report on a 1 V lithium sulfide electrode with noteworthy performance, demonstrating that sulfide-based electrodes may merit further exploration.

## Graphical abstract

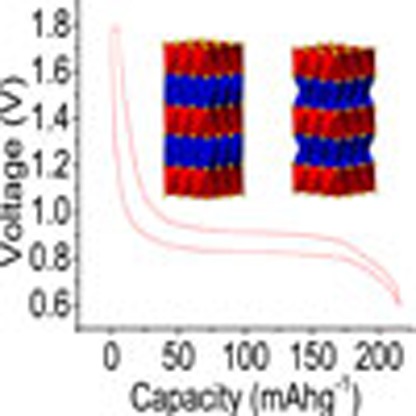

In order to avoid the potential danger of Li plating^1^,^2^ on the widely used graphite anodes^3^,^4^,^5^ in Li-ion batteries, which intercalate Li at 0.1 V versus Li^+^/Li, important efforts have been made to identify anodes operating at 1 V (refs 6, 7, 8, 9), including organic intercalation compounds^10^ and conversion reactions^11^,^12^. In the former case, the low density inherent in molecular intercalation electrodes (typically ∼1.6 g cm^−3^) (ref. 13) leads to low volumetric capacities. Volumetric energy density is an important parameter for future Li-ion batteries. The majority of conversion reactions exhibit large polarization (often in excess of 1 V), and voltages above 1 V on Li removal (discharge when used as an anode) are observed frequently^7^,^14^. In contrast, inorganic intercalation electrodes operate by a well-established mechanism, without the drastic changes that accompany conversion reactions, without involving nanoparticles and with higher densities than organic intercalation compounds.

Li can be intercalated into the layered compound LiVO_2_ (cubic close packed structure) but at the very low voltage of <0.1 V (refs 15, 16, 17). In contrast, Li intercalation into the early transition metal sulfides, LiMS_2_, M=V, Ti, occurs in the region of 1 V (M^2+/3+^) and these materials have been explored previously, in pioneering studies, as possible anodes for Li-ion batteries^18^,^19^,^20^.

However, previous work on LiVS_2_ has shown that following Li intercalation, the subsequent Li extraction (corresponding to discharge when the LiVS_2_ is used as an anode in a Li-ion battery) occurs at 1.3 V even at low rates and increases further with rate^18^,^20^. LiVS_2_ is therefore not a true 1 V anode. Li intercalation into LiTiS_2_ occurs at a lower voltage of 0.5 V, and exhibits very poor capacity retention on cycling, the capacity reducing to only 120 mAhg^−1^ after just five cycles. LiTiS_2_ also exhibits a massive 1st cycle irreversible loss of capacity of over 180 mAhg^−1^ (ref. 18). We investigate the complete solid solution range Li(V_1-*x*_Ti_*x*_)S_2_ and find that the 50/50 composition Li(V_0.5_Ti_0.5_)S_2_ delivers a significant improvement in properties/performance over other compositions, including the end members. A total of 50% replacement of V by Ti is sufficient to lower the potential (by lowering the Fermi level in the *d*-states) even on deintercalation (charge) below 1 V (0.9 V), but not to the extent of LiTiS_2_ (where the consequence is massive irreversible capacity loss). The irreversible loss of capacity for Li(V_0.5_Ti_0.5_)S_2_ on the 1st cycle is only 42 mAhg^−1^ at C/2 (compared with 180 mAhg^−1^ for LiTiS_2_). Furthermore, the polarization on the first cycle is half that of LiVS_2_ at the same rate. Li(V_0.5_Ti_0.5_)S_2_ exhibits a reversible capacity of 220 mAhg^−1^ at C/2 (99% of the theoretical capacity) dropping only marginally to 200 mAhg^−1^ at 3C. The point of the paper is not to claim that the Li(V_0.5_Ti_0.5_)S_2_ material reported here is a commercially viable anode, with all practical problems solved. Rather it is to show that LiMS_2_ based electrodes are not limited to the relatively poor performance (drastic capacity fading, large 1st cycle irreversible capacity and large polarization) reported previously and that sulfide based electrodes may merit further exploration in the future.

## Results

### Li(V_1-*x*_Ti_*x*_)S_2_ solid solution

**Figure 1:**
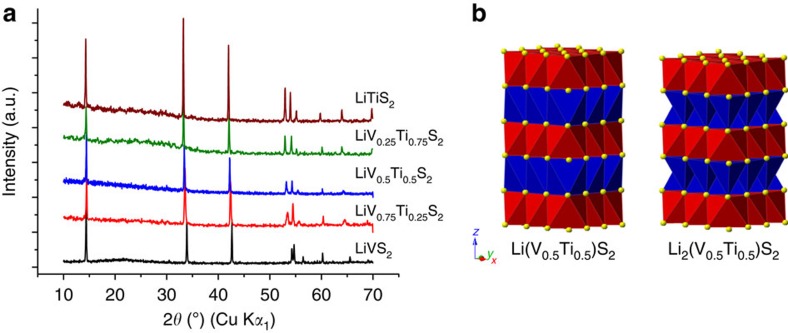
Structures of Li(V_1-*x*_Ti_*x*_)S_2_ and Li_2_(V_0.5_Ti_0.5_)S_2_. (**a**) Powder X-ray diffraction patterns of as-prepared Li(V_1-*x*_Ti_*x*_)S_2_. (**b**) Crystal structures of Li(V_0.5_Ti_0.5_)S_2_ and Li_2_(V_0.5_Ti_0.5_)S_2_. Red octahedra—MS_6_, blue polyhedra—LiS_*x*_ and yellow spheres—sulfur.

The range of compositions across the Li(V_1-*x*_Ti_*x*_)S_2_ solid solution were synthesized as described in the Methods section, where the electrode fabrication and characterization methods are also described. The powder X-ray diffraction patterns are shown in Fig. 1a and correspond to the LiMS_2_ structure, composed of hexagonal close packed S^2−^ with alternate sheets of octahedral sites occupied by Li and M cations, Fig. 1b. A comparison of powder X-ray patterns for Li(V_0.5_Ti_0.5_)S_2_ and the blend of 50 wt% LiVS_2_ and 50 wt% LiTiS_2_ is shown in Supplementary Fig. 1, confirming that the solid solution has been successfully synthesized. Li intercalation into this structure would require Li^+^-occupying tetrahedral sites that share faces with Li in octahedral sites, and is therefore energetically unfavourable. Instead, upon intercalation, all the Li^+^ ions in the octahedral sites in LiMS_2_ are displaced to the neighboring tetrahedral sites in the alkali metal layers, as there are twice as many tetrahedral as octahedral sites the intercalating Li can be incorporated up to a theoretical maximum of Li_2_MS_2_ (ref. 18).

**Figure 2:**
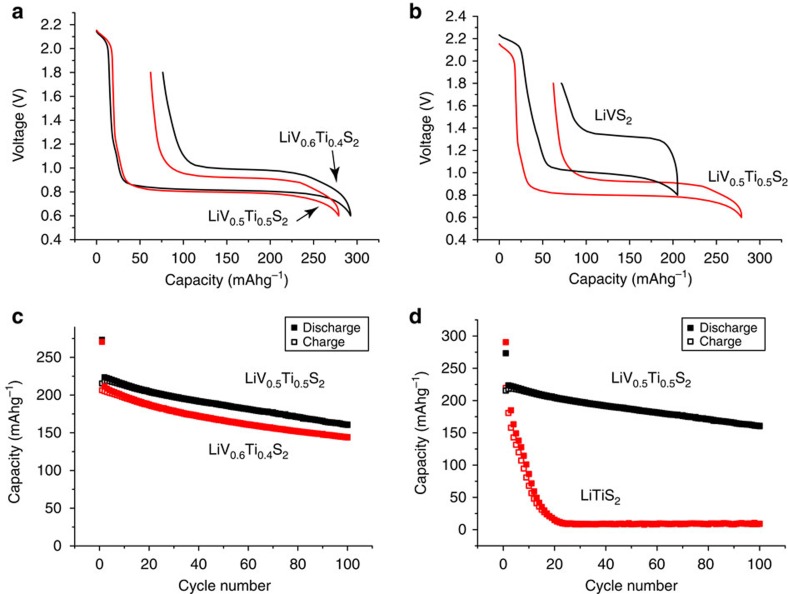
Comparisons between Li(V_0.5_Ti_0.5_)S_2_ and other members of the Li(V_1-*x*_Ti_*x*_)S_2_ solid solution. (**a**,**b**) show the variation of potential with state-of-charge on the 1st cycle for Li(V_0.5_Ti_0.5_)S_2_ and Li(V_0.6_Ti_0.4_)S_2_, and LiVS_2_ respectively. (**c**,**d**) show the variation of capacity with cycle number for Li(V_0.5_Ti_0.5_)S_2_ and Li(V_0.4_Ti_0.6_)S_2_, and LiTiS_2_ respectively, Closed squares correspond to intercalation and open squares to deintercalation. All data were collected at a rate of 100 mAg^−1^.

Li(V_0.5_Ti_0.5_)S_2_ was found to be the best performing of all the Li(V_1-*x*_Ti_*x*_)S_2_ materials. Materials with more vanadium displayed increased polarization and deintercalation takes place above 1 V, comparisons with Li(V_0.6_Ti_0.4_)S_2_ and LiVS_2_ are presented in Fig. 2a,b. Increasing the titanium content beyond 50% results in a decrease in capacity and increase in irreversible capacity on the 1st cycle, as is demonstrated by the Li(V_0.4_Ti_0.6_)S_2_ and LiTiS_2_ data in Fig. 2c,d.

### The electrochemical properties of Li(V_0.5_Ti_0.5_)S_2_

**Figure 3:**
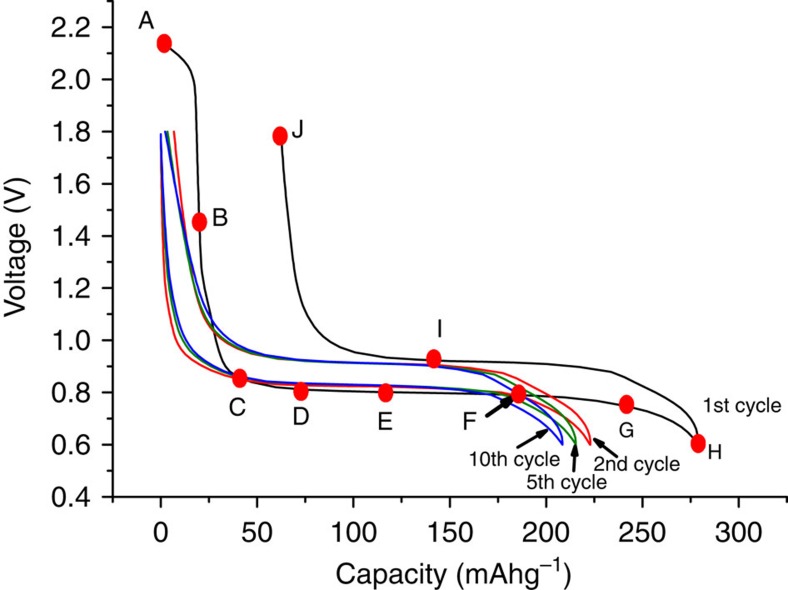
Variation of potential (versus Li^+^/Li) with state of charge for Li(V_0.5_Ti_0.5_)S_2_. Rate 100 mAg^−1^. Letters correspond to various states of charge on the 1st cycle, see also Fig. 5.

The charge–discharge curves for Li(V_0.5_Ti_0.5_)S_2_ are shown in Fig. 3. The capacity on cycling is 220 mAhg^−1^ (2nd cycle) with the plateau on deintercalation (corresponding to the potential of ;the anode on discharge in a Li-ion cell) being located at 0.9 V. The polarization (separation between discharge and charge plateaus) is small, ∼100 mV (rate=C/2). The load curves are invariant on subsequent cycling, save for a slow reduction in capacity. Concerning the first cycle, there is an initial capacity of 20 mAhg^−1^ at ∼2.1 V, associated with the M^4+/3+^ redox couple and consistent with a small degree of lithium deficiency in the as-prepared material, Li_0.92_(V_0.5_Ti_0.5_)S_2_, see refinement of structure, Supplementary Table 1. The main difference between the 1st and subsequent cycles is an additional, irreversible, capacity of ∼40 mAhg^−1^on cycle 1, Fig. 3, and is associated with reduction of the electrolyte. Electrolyte reduction/solid electrolyte interphase (SEI) layer formation has been observed previously for LiVS_2_ and LiTiS_2_ (ref. 18) and is discussed later for Li(V_0.5_Ti_0.5_) S_2_.

**Figure 4:**
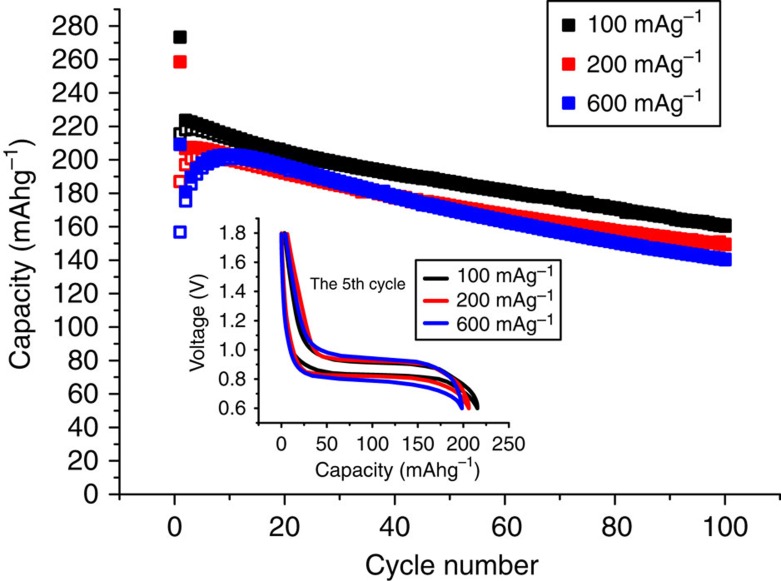
Variation of capacity with cycle number for Li(V_0.5_Ti_0.5_)S_2_. Closed squares correspond to intercalation and open squares to deintercalation. Inset shows load curves as a function of rate on the 5th cycle.

The capacity as a function of cycle number for several different rates is shown in Fig. 4. The capacity on cycling is well maintained with increasing rate, for example, dropping by only 6% on increasing the rate from C/2 to 3C at cycle 10. The capacities on intercalation/de-intercalation during the first few cycles are affected by the irreversible capacity (on cycle 1) and at higher rates by an increase in capacity on cycling, something not infrequently seen in intercalation electrodes at high rates and often related to changes in the composite electrode structure within the first few cycles^21^,^22^. After the irreversible capacity loss on cycle 1, the charge/discharge efficiency improves rapidly over the first few cycles at all rates, Fig. 4. Overall the capacity retention on cycling after the first few cycles corresponds to a loss of ∼0.6 mAhg^−1^ per cycle. The origin of the capacity fading on cycling lies in the SEI layer formation and is discussed below.

### The mechanism of intercalation into Li(V_0.5_Ti_0.5_)S_2_

**Figure 5:**
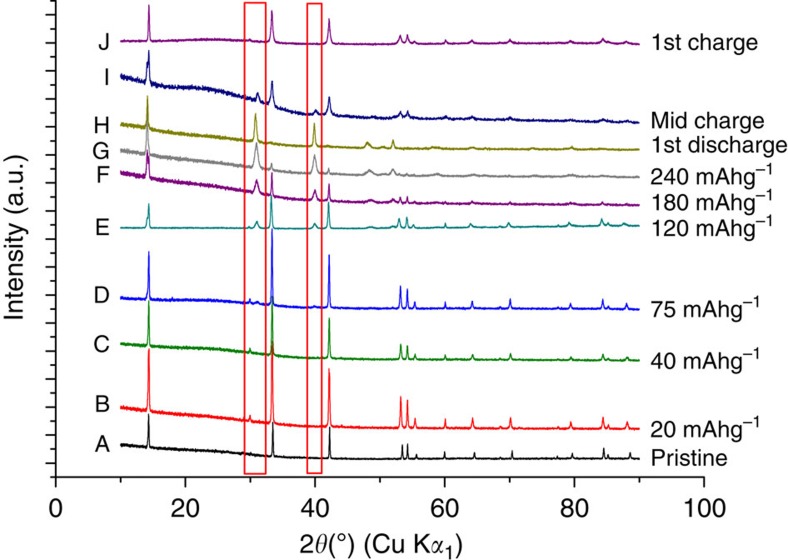
Powder X-ray diffraction patterns of Li(V_0.5_Ti_0.5_)S_2_ at various states of intercalation and deintercalation. Letters correspond to points on the 1st cycle in Fig. 3. Highlighted regions show the presence of Li_2_(V_0.5_Ti_0.5_)S_2_ phase.

Given the change in properties/performance exhibited by the Li(V_0.5_Ti_0.5_)S_2_ solid solution compared with the stoichiometric LiVS_2_ and LiTiS_2_ end members, it is important to ascertain if lithium intercalation into Li(V_0.5_Ti_0.5_)S_2_ operates by the same mechanism. The results below show that the improved properties of Li(V_0.5_Ti_0.5_)S_2_, are obtained despite a similar two-phase intercalation process and all three materials forming a SEI layer due to electrolyte degradation. Considering the structure of Li(V_0.5_Ti_0.5_)S_2_ and its evolution on intercalation/deintercalation and cycling, joint refinements were carried out on the as-prepared material using the powder X-ray diffraction data in Supplementary Fig. 2 and the neutron diffraction data in Supplementary Fig. 3. Refined parameters are given in Supplementary Table 1. The refined composition, Li_0.92_(V_0.5_Ti_0.5_)S_2_ is in good agreement with the capacity associated with the intercalation at 2.1 V (0.08 Li corresponds to 18 mAhg^−1^). There is no evidence of site exchange between Li and V/Ti, that is, the structure remains layered, and no evidence of V/Ti long-range order. The V/Ti ratio refined to 1:1 within errors. The variation of structure with Li insertion/removal is shown in Fig. 5. The diffraction patterns were collected at the points on the load curve shown in Fig. 3. Initially, on intercalation Li fills the vacant octahedral sites in the Li layers of the as-prepared Li_0.92_(V_0.5_Ti_0.5_)S_2_, accounting for the first 18–20 mAhg^−1^ (between A and B in Fig. 3), this results in an expansion in ***a*** and ***c***, from ***a***=3.4247(1) Å and ***c***=6.1582(2) Å to ***a***=3.4377(4) Å and ***c***=6.1591(2) Å. There is no change in the diffraction patterns between points B and C. Thereafter, Li intercalation is a 2-phase process, in accord with the plateau in Fig. 3, in which the as-prepared phase, Li(V_0.5_Ti_0.5_)S_2_ is replaced continuously by Li_2_(V_0.5_Ti_0.5_)S_2_. On lithium extraction the 2-phase process is reversed, associated with the plateau on charge, as seen at point I in Fig. 3. Two phase refinements at points D–G and at I are well described by a mixture of the two end members with the compositions Li(V_0.5_Ti_0.5_)S_2_ and Li_2_(V_0.5_Ti_0.5_)S_2_ and the patterns at A, B, C, H and J by single phases of the two end members. A cyclic voltammogram collected on a 3-electrode cell with the Li(V_0.5_Ti_0.5_)S_2_ as the working electrode is shown in Supplementary Fig. 4 and exhibits one oxidation peak around 1 V and one reduction peak at 0.7 V, its shape is consistent with a 2-phase process, in accord with the powder X-ray diffraction results^23^. The above results show that the mechanism of Li intercalation into Li(V_0.5_Ti_0.5_)S_2_ is essentially similar to that of LiVS_2_ (ref. 18).

**Figure 6:**
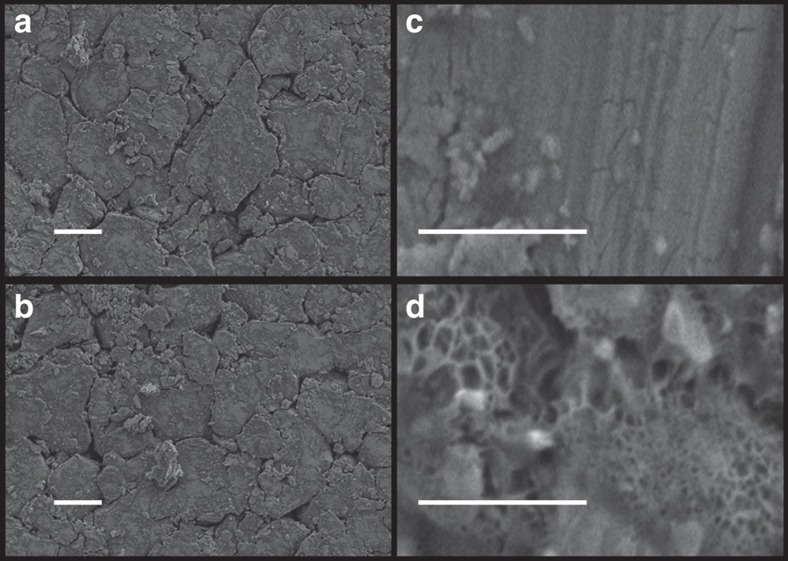
SEM images. (**a**) Pressed pristine Li(V_0.5_Ti_0.5_)S_2_ electrode and (**b**) Li(V_0.5_Ti_0.5_)S_2_ electrode after 5 cycles. (**c**) Pristine Li(V_0.5_Ti_0.5_)S_2_ electrode and (**d**) Li(V_0.5_Ti_0.5_)S_2_ electrode after 1st discharge (intercalation) showing SEI layer formation on the surface of the electrode. Scale bar, (**a**,**b**) 10 μm, (**c**,**d**) 1 μm.

The refined **a** and **c** lattice parameters for Li_1_(V_0.5_Ti_0.5_)S_2_ and Li_2_(V_0.5_Ti_0.5_)S_2_ are respectively ***a***=3.4377(4) Å, ***c***=6.1591(2) Å and ***a***=3.7894(7) Å, ***c***=6.229(2) Å. The volume expansion on intercalation is 23% yet the phase transformation is facile. The expansion along ***a*** is associated with the elongation of the (Ti/V)-S bond as formally (Ti/V)^3+^ is reduced to (Ti/V)^2+^. As the *d*-bands in sulfides are relatively wide compared with oxides, the electrons are well delocalized across the (Ti/V)-S_2_ slabs. The ***c***-axis expansion requires energy to increase the separation of the slabs. The small ***c***-axis expansion, only 1.5%, may be responsible for the facile nature of the 2-phase intercalation reaction that exhibits a small polarization and excellent reversibility. Powder X-ray diffraction (PXRD) data collected on cycling, Supplementary Fig. 5 shows that the 2-phase intercalation/de-intercalation mechanism continues to operate. There is no change in lattice parameters with cycling. SEM images of electrodes were collected before and after cycling, Fig. 6a,b. The average particle size of Li(V_0.5_Ti_0.5_)S_2_ is 20 μm and remains so on cycling. All of these results indicate that the material is stable on cycling.

### SEI layer formation and capacity fade

Electrolyte reduction and SEI layer formation have been reported for LiVS_2_ and LiTiS_2_, where it was inferred only from the electrochemistry^18^. Of the ∼40 mAhg^−1^ of irreversible capacity on the first cycle for Li(V_0.5_Ti_0.5_)S_2_, Fig. 3, the 20 mAhg^−1^ of capacity between B and C may be assigned to electrolyte reduction, as there is no change in the lattice parameters. Beyond C, the Rietveld refinements of the 2-phase mixtures of Li(V_0.5_Ti_0.5_)S_2_ and Li_2_(V_0.5_Ti_0.5_)S_2_ along the plateau provide the ratios of the two phases, which in turn gives the amount of lithium and hence charge that has been inserted into the structure. These values are presented in Table 1 along with the values for the total charge passed minus the 20 mAhg^−1^ at the beginning of discharge associated with the M^4+/3+^ redox couple. There is almost no difference in these values across the plateau, indicating that no significant electrolyte reduction occurs between C and G in Fig. 3. The remainder of the electrolyte reduction occurs beyond point G, in accord with the slow downturn in the potential. Extending the cut-off potential to lower values increases this electrolyte reduction and hence irreversible capacity significantly, which is why lower voltage anodes are less attractive. The amount of true intercalation capacity derived from the refinement of the PXRD data is consistent with the theoretical capacity for Li(V_0.5_Ti_0.5_)S_2_ (222 mAhg^−1^) and the observed capacity on the 2nd cycle, Fig. 3. This demonstrates that the additional (irreversible) capacity on cycle 1 is due primarily to electrolyte reduction.

It is important to recall that the composite electrodes contain 10% super S carbon, and this will account for some of the observed electrolyte reduction (irreversible capacity). Cells were constructed with the same loading of Li(V_0.5_Ti_0.5_)S_2_ per unit area as in Fig. 3, but without super S carbon, the comparison between the two cells is shown in Supplementary Fig. 6. The load curve without super S shows virtually no capacity between 0.93 and 1.3 V, suggesting that the ∼12 mAhg^−1^ loss observed between those voltages for the electrode containing Super S is associated with electrolyte reduction on the carbon surface. The capacity between 0.93 V and point C, 8 mAhg^−1^, could be due to reduction of the electrolyte on the surface of Li(V_0.5_Ti_0.5_)S_2_ as it is present in the electrode with and without carbon. It might also arise from a very small amount of intercalation, corresponding to *x*=0.03, too small to be seen in the X-ray data. The irreversible capacity loss occurring at the lower voltage beyond the plateau, <0.7 V, appears also due to a combination of carbon and Li(V_0.5_Ti_0.5_)S_2_, since it increases when super S is present. Overall, Supplementary Fig. 6 shows that of the irreversible capacity observed in Fig. 3, only 25 mAhg^−1^ arises from electrolyte reduction on the Li(V_0.5_Ti_0.5_)S_2_ surface that is, 11% of the reversible capacity, the rest is due to reduction on the surface of the super S.

**Figure 7:**
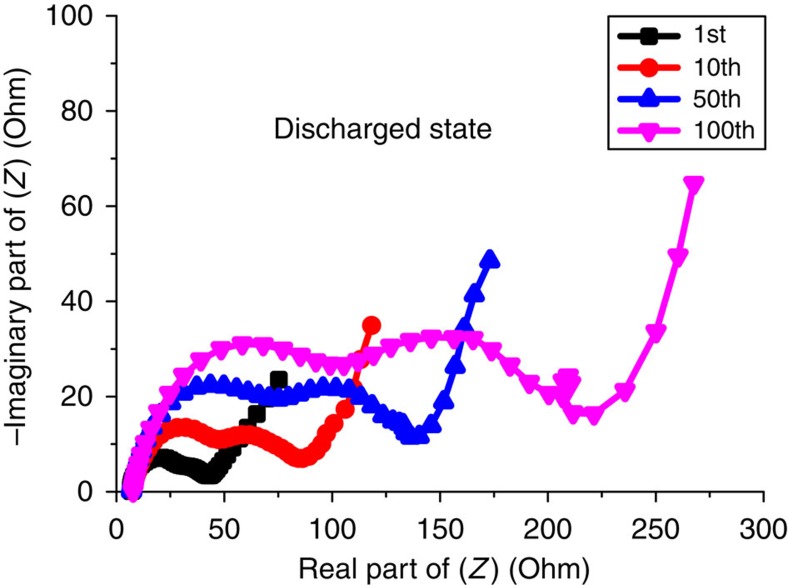
AC impedance spectra. AC impedance of the Li(V_0.5_Ti_0.5_)S_2_/electrolyte interface collected at the end of the 1st, 10th,50th and 100th cycles using 3-electrode cells.

**Figure 8:**
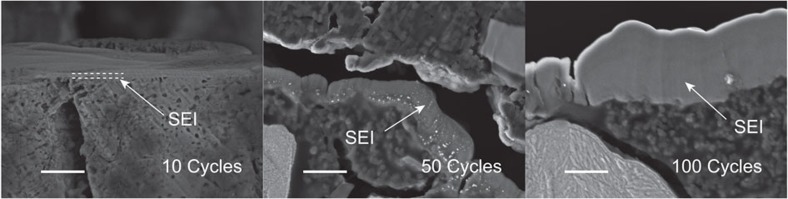
Cross-sectional SEM images. Cross-sectional SEM images of the Li(V_0.5_Ti_0.5_)S_2_ electrode collected at the end of the 10th, 50th and 100th cycles. Scale bar, 1 μm.

To explore the SEI layer formation as a function of cycling, alternating current (AC) impedance spectroscopy and SEM (scanning electron microscopy) images were collected from cycles 1 to 100, Figs 7 and 8. The AC impedance data, from 3-electrode cells, show two semicircles, as is typical for the electrode/electrolyte interface^24^. The interfacial impedance grows continuously with cycle number indicative of a growing SEI layer. This was confirmed by examination of the SEM images, Fig. 8, where the SEI layer is seen to grow on the electrode particles from several nanometers at cycle 10 up to >1 μm at cycle 100. The growing SEI layer is consistent with the capacity fading observed on cycling. Confirmation that the capacity fading is not intrinsic to the material but is due to the SEI layer growth was provided by the powder X-ray diffraction patterns at the 25th cycle, Supplementary Fig. 5, which showed the crystal structure is preserved and the 2-phase reaction remains on cycling. We have used standard LP30 electrolyte, but this is optimized for graphite and not for sulfide electrodes operating near 1 V. A detailed investigation of alternative electrolytes, electrolyte additives and of surface coatings on the sulfide may be able to reduce further the irreversible capacity on the 1st cycle even below the 11% associated with reduction on the Li(V_0.5_Ti_0.5_)S_2_ itself, and to reduce significantly the capacity fading on cycling.

### Li(V_0.5_Ti_0.5_)S_2_ in a full lithium-ion cell

**Figure 9:**
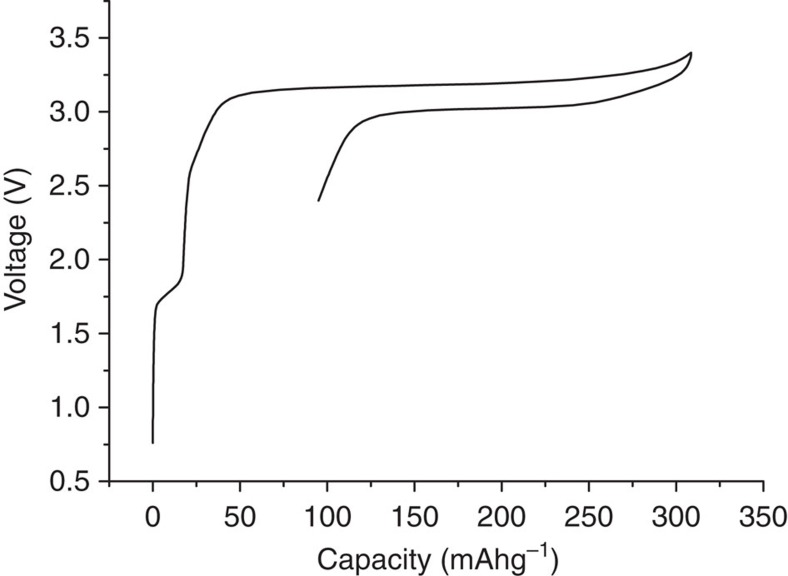
Variation of potential with state of charge for the Li(V_0.5_Ti_0.5_)S_2_/LiCoO_2_ cell. Rate 100 mAg^−1^ of anode, cells were cycled between 3.4 V and 2.4 V.

Li(V_0.5_Ti_0.5_)S_2_ was incorporated as the anode in a full lithium-ion cell with a LiCoO_2_ cathode. The cell was constructed such that the overall capacity was anode limited, in order to show the performance of the latter, see Methods section. First charge capacity, corresponding to insertion of lithium into Li(V_0.5_Ti_0.5_)S_2_, exhibits a larger irreversible capacity than was observed when the counter electrode was lithium metal (∼75 mAhg^−1^ as opposed to 42 mAhg^−1^), see Fig. 9. In full cells the anode potential can reach lower values on charge than is the case for a lithium counter electrode. If this happens there will be a higher irreversible capacity, as observed here. The overall capacity on the first charge in Fig. 9 is 310 mAhg^−1^ compared with 278 mAhg^−1^ for the cell with a Li counter electrode, the difference is the same as the additional irreversible capacity (75–42 mAhg^−1^), consistent with the additional irreversible capacity in Fig. 9. Lowering the voltage cutoff below 3.4 V seen in Fig. 9 leads to a loss of reversible capacity on the first and subsequent cycles. The discharge capacity is ∼215 mAhg^−1^, just below the theoretical capacity; this reduces to just over 180 mAhg^−1^ by the 10th cycle, see Supplementary Fig. 7. The purpose of this experiment was simply to show that a full cell can be constructed and cycled using the new anode.

Sulfides are used in a variety of applications ranging from pigments^25^ to solar cells^26^, they are not exotic materials. Although the sulfides here are synthesized in sealed tubes for convenience in the lab, this is not ubiquitous for sulfides, other methods can be used^27^,^28^. V is not the lowest cost element but several V based electrodes are under investigation and have been reported, for example, V_2_O_5_, Na_*x*_VO_2_, LiVO_2_, and of course V redox-flow batteries are in use and continue to be studied^15^,^29^,^30^,^31^. The materials we report showed no change in their powder X-ray diffraction patterns after 12 h exposure to ambient air, Supplementary Fig. 8. After 15 h a small peak begins to appear at 11° in 2*θ*, Supplementary Fig. 8 and grows with time, showing that the sulfides are air sensitive as expected. The widely used LiFePO_4_ cathode material is also not stable in air and is packed in sealed containers after synthesis for shipping. A similar approach could be taken with Li(V_0.5_Ti_0.5_)S_2_. We suggest that the relatively slow rate of reaction in air is likely due to the formation of a thin oxide layer that slows decomposition.

## Discussion

An intercalation anode, Li(V_0.5_Ti_0.5_)S_2_, is reported that operates at 0.9 V versus Li^+^/Li (56% lower voltage than Li_4_Ti_5_O_12_ refs 32, 33, 34), with low polarization, 100 mV, a capacity of 220 mAhg^−1^ at C/2 on cycling, corresponding to 99% of theoretical capacity, good rate capability, 205 mAhg^−1^ at 1C and 200 mAhg^−1^ at 3C. The volumetric capacity is 740 mAhcm^−3^, comparable to the reversible volumetric capacity of graphite (600–700 mAh cm^−3^)^35^, but at a potential (0.9 V) that avoids Li plating. As such, this material delivers significantly better properties/performance than the previously studied end-members, LiVS_2_ (the voltage of which is >1 V versus Li^+^/Li) and LiTiS_2_ (which exhibits a very large irreversible capacity loss on the 1st cycle of 180 mAhg^−1^). Electrolyte reduction on Li(V_0.5_Ti_0.5_)S_2_ during the first cycle accounts for only 11% of the first cycle capacity. Performance is better than previously reported 1 V anodes, to the best of our knowledge. Future work should focus on reducing the 1st cycle irreversible capacity while forming a robust SEI layer minimizing further electrolyte reduction, by examining lowering the amount of conductive additive in the electrode and investigating electrolyte additives or seeking alternative electrolytes to those optimized for graphite anodes.

## Methods

### Synthesis

Li(V_0.5_Ti_0.5_)S_2_ was synthesized from Li_2_S (Aldrich, 99.9%), Ti powder (Aldrich, 99.98%), V powder (Aldrich, 99.5%) and S powder (Aldrich, 99.98%). Appropriate ratios of starting materials were mixed and ground together in an Ar filled MBraun glovebox. The mixture was then placed in a graphite crucible, which was in turn placed in a quartz tube. The quartz tube was subsequently sealed under vacuum. The reactants were heated at 1,050 °C for 72 h before being quenched to room temperature.

### Electrochemical measurements

Composite electrodes were fabricated using the active material, super S carbon and Kynar Flex 2801 (a co-polymer based on polyvinylidene difluoride) binder in a mass ratio of 80:10:10. The mixture was then cast on copper foil using tetrahydrofuran as the solvent. The loading of active material was ∼6–8 mg cm^−2^. Electrochemical cells consisting of a Li(V_0.5_Ti_0.5_)S_2_ composite electrode, a lithium metal counter electrode and a glass microfiber GF/F separator (WhatmanTM) saturated with electrolyte, a 1 M solution of LiPF_6_ in ethylene carbonate–dimethyl carbonate 1:1 ((v/v) (BASF)), were constructed. Li-ion cells were constructed similarly with composite LiCoO_2_ electrodes (LiCoO_2_, Super S and Kynar Flex 2801 in a mass ratio of 80:10:10) replacing the lithium metal counter. Celgard monolayer polypropylene separator was used in addition to the glass microfiber separator. All handling was carried out in an Ar filled MBraun glovebox. Electrochemical measurements were conducted using a Maccor series 4,200 battery tester. Cyclic voltammetry and AC impedance were conducted on 3-electrode cells using a VMP3 electrochemical workstation (Biologic).

### Structural analysis

Powder X-ray diffraction patterns were obtained using a Stoe STADI/P diffractometer employing CuK*α*_1_ radiation operating in transmission mode with the samples sealed in 0.2 mm capillaries, expect for the experiments on air sensitivity, which were carried out in air. Time-of-flight powder neutron diffraction data were collected on the POLARIS high-intensity; medium resolution instrument at ISIS, Rutherford Appleton Laboratory (UK) with samples sealed in 2 mm quartz capillaries. The structures were refined by the Rietveld method using TOPAS Academic^36^. Samples with different Li amounts for X-ray diffraction were prepared electrochemically. After cycling, cells were transferred to an argon-filled glove box before opening and active material removed. The electrodes were then rinsed with a small amount of dry dimethyl carbonate to remove residual electrolyte. They were left under dynamic vacuum overnight to ensure all solvent had evaporated before measurements were collected. SEM studies were carried out using a Carl Zeiss Merlin instrument. Electrodes for cross-sectional imaging were rotary-etched in a Gatan Precision Etching Coating System (PECS 682).

## Additional information

**How to cite this article:** Clark, S. J. *et al.* Li(V_0.5_Ti_0.5_)S_2_ as a 1 V lithium intercalation electrode. *Nat. Commun.* 7:10898 doi: 10.1038/ncomms10898 (2016).

## Supplementary Material

Supplementary Information Supplementary Figures 1-8 and Supplementary Table.

## Figures and Tables

**Table 1:** Comparison between capacity observed electrochemically and intercalated capacity derived from X-ray refinements.

Point	Capacity from load curve minus the M^4+/3+^ couple (mAhg^−1^)	Capacity of Li intercalated into Li(V_0.5_Ti_0.5_)S_2_ from X-ray analysis (mAhg^−1^)	Difference (mAhg^−1^)
B	0	0	0
C	20	0	20
D	50	28	22
E	95	72	23
F	155	133	22
G	215	195	20
H	258	216	42

Letters correspond to the points on the 1st cycle in Fig. 3.
