# Supplementary material for: Li(V0.5Ti0.5)S2 as a 1 V lithium intercalation electrode
Source: Nat Commun. 2016 Mar 21;7:10898. doi: 10.1038/ncomms10898 (PMC4802118; doi:10.1038/ncomms10898)
Supplement: Supplementary Information — Supplementary Figures 1-8 and Supplementary Table. [file ncomms10898-s1.pdf]

Supplementary Information

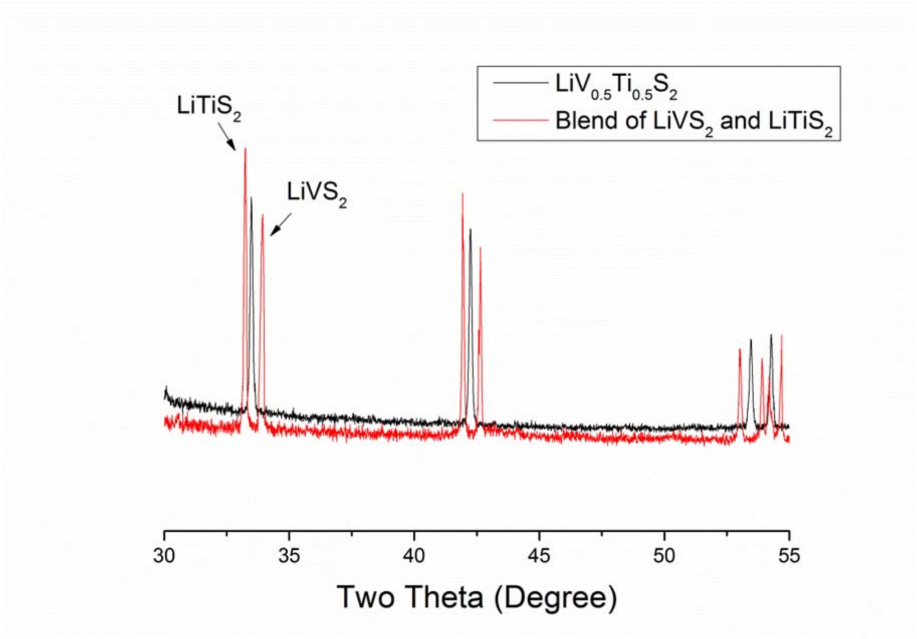

Supplementary Figure 1: Comparison of powder X-ray patterns for $Li(V_{0.5}Ti_{0.5})S_2$ and the blend of 50 wt% $LiVS_2$ and 50 wt% $LiTiS_2$

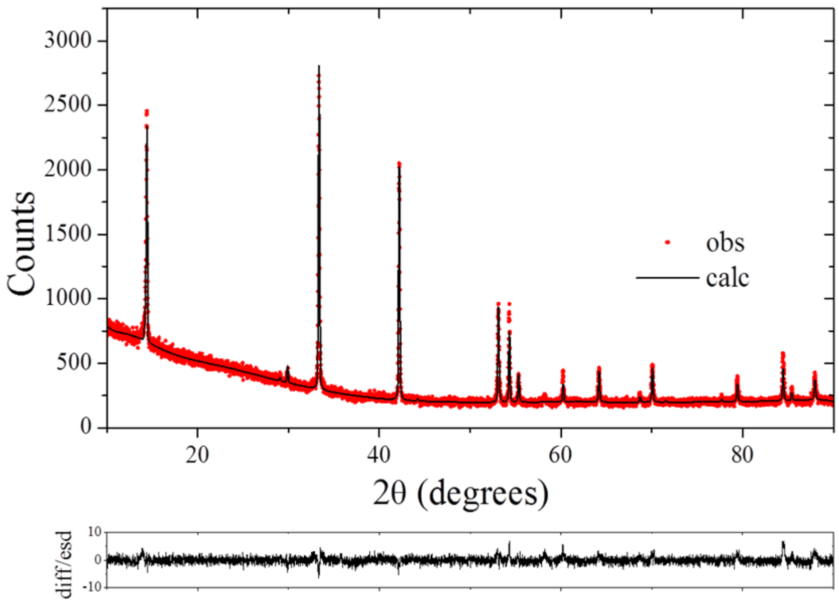

Supplementary Figure 2: Powder X-ray diffraction for $Li_{0.92}(V_{0.5}Ti_{0.5})S_2$ Experimental data (red dots) compared with the Rietveld refined profile (black line) and the difference/esd curve (lower line) for $Li_{0.92}$ $(V_{0.5}Ti_{0.5})S_2$.

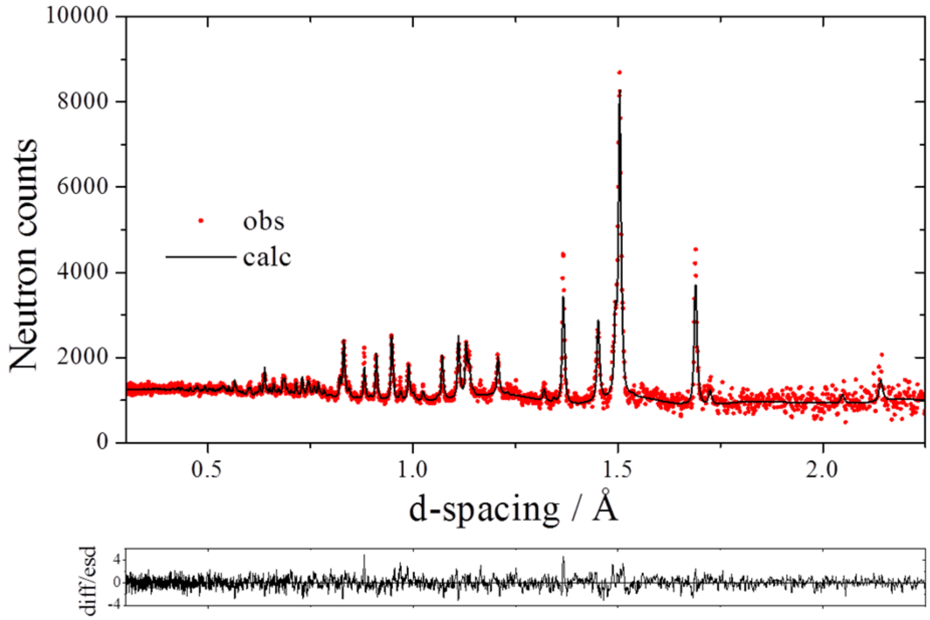

Supplementary Figure 3: Powder Neutron diffraction for $Li_{0.92}(V_{0.5}Ti_{0.5})S_2$ Experimental data (red dots) compared with the Rietveld refined profile (black line) and the difference/esd curve (lower line) for $Li_{0.92}$ $(V_{0.5}Ti_{0.5})S_2$.

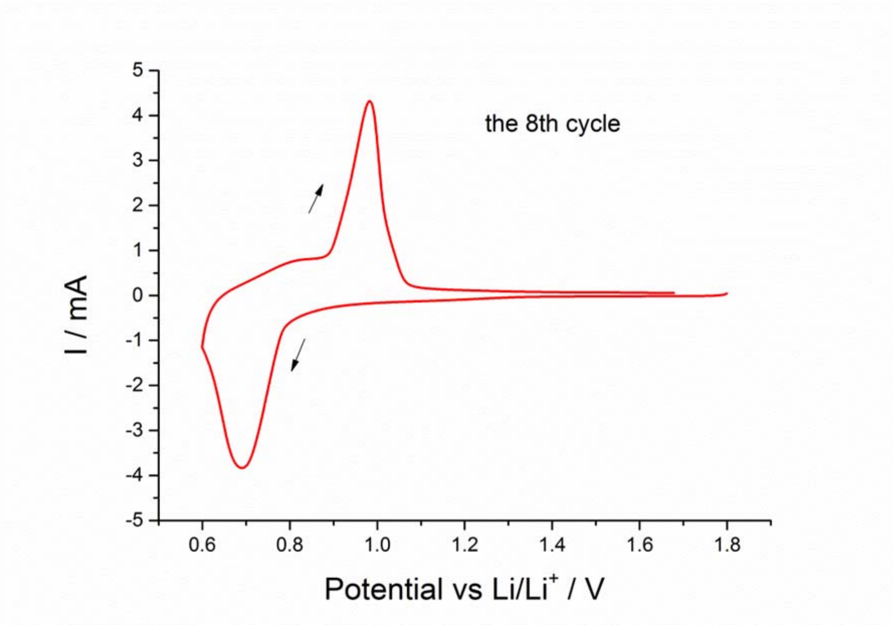

Supplementary Figure 4: Cyclic voltammogram collected on the 8th cycle Scan rate 0.2mVs^{-1}.

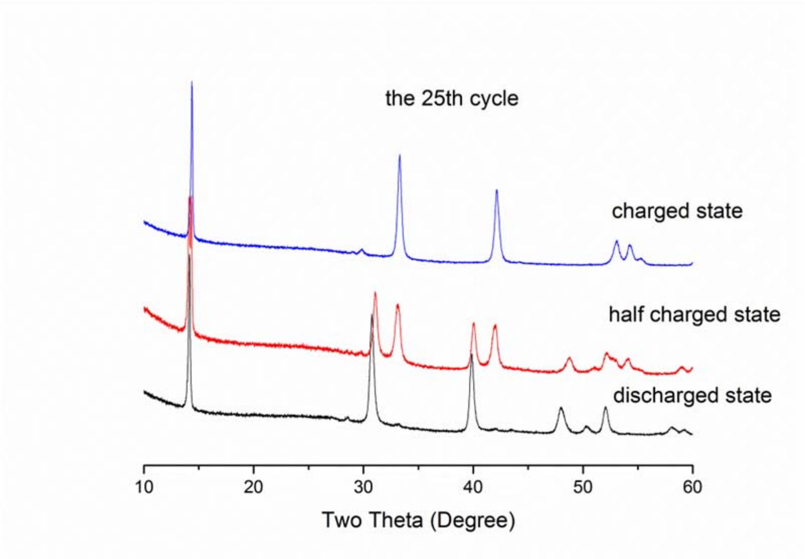

Supplementary Figure 5: PXRD patterns collected on the 25th cycle

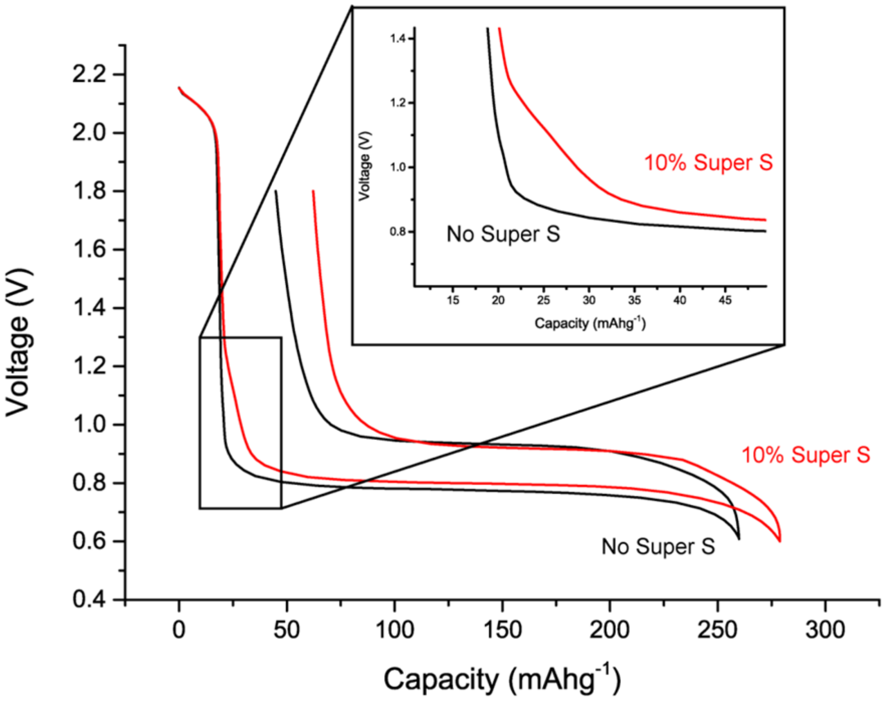

Supplementary Figure 6: Variation of potential with state-of-charge for the 1st cycle in $Li(V_{0.5}Ti_{0.5})S_2$ using 0% Super S carbon and 10% Super S carbon Rate 100 mAg^{-1}. Insert shows expanded region between 1.4 and 0.85 V.

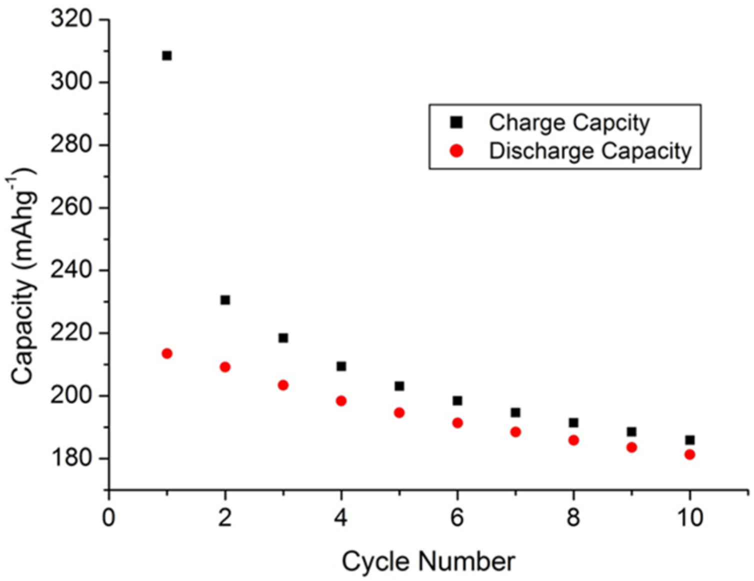

Supplementary Figure 7: Variation of capacity with cycle number for $Li(V_{0.5}Ti_{0.5})S_2/LiCoO_2$ full cell

Rate $100mAg^{-1}$, black and red dots correspond to intercalation and deintercalation respectively.

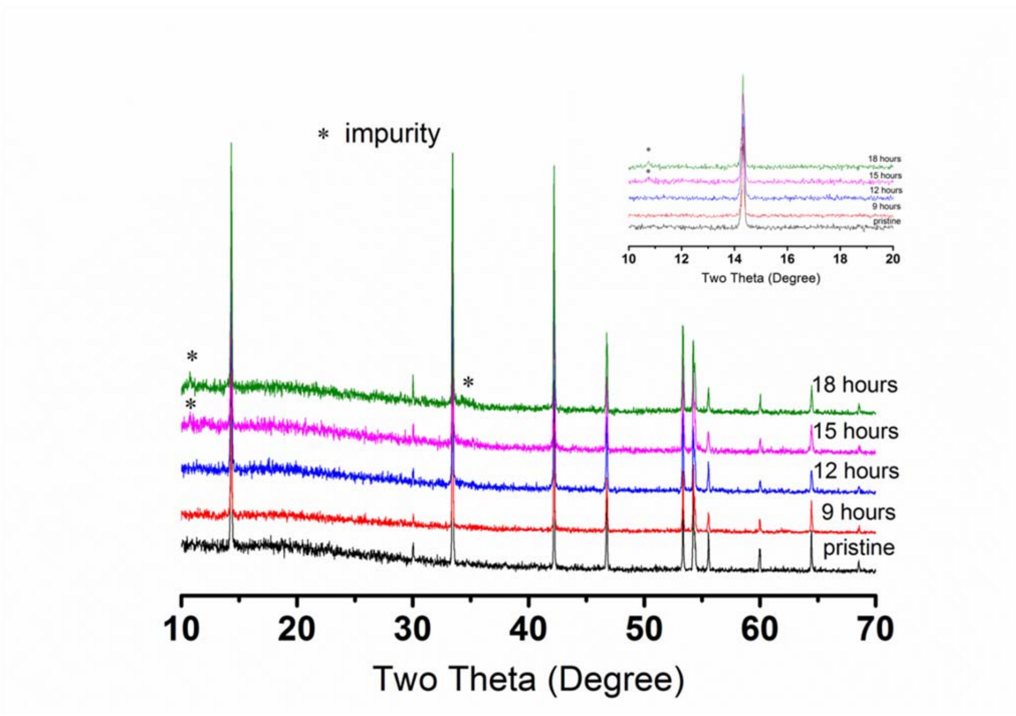

Supplementary Figure 8a: Comparison of PXRD patterns collected on $Li(V_{0.5}Ti_{0.5})S_2$ after different exposure times in air

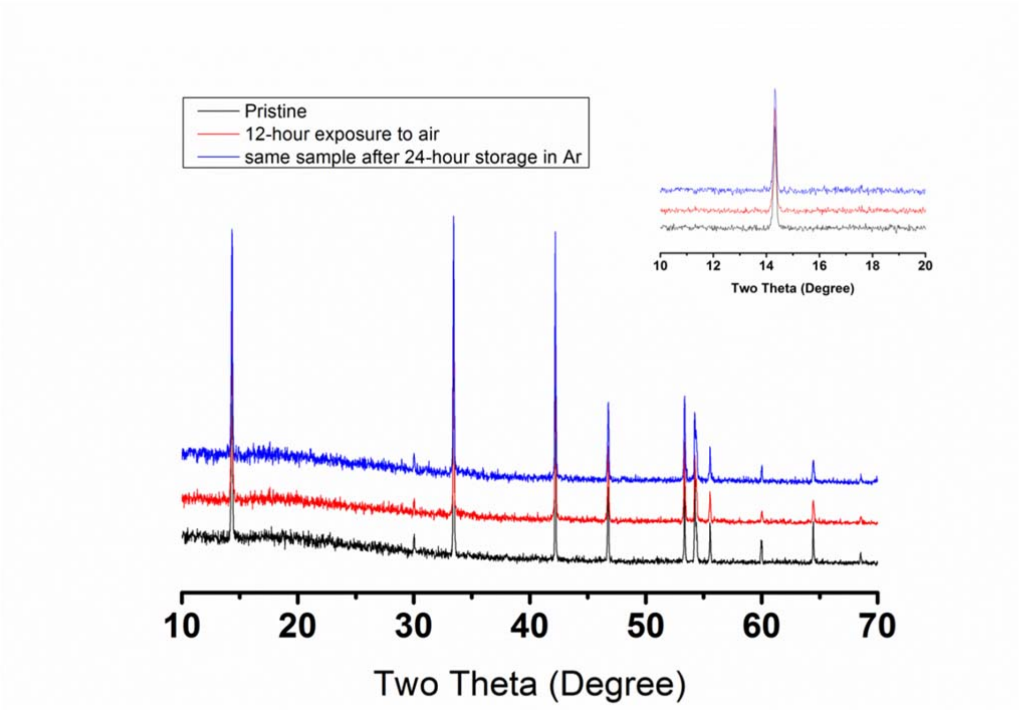

Supplementary Figure 8b: Comparison of PXRD patterns collected on pristine $Li(V_{0.5}Ti_{0.5})S_2$, after 12-hour exposure to air then 24-hour storage in Argon

Supplementary Table 1: Refined parameters for as-prepared $Li_{0.92}(V_{0.5}Ti_{0.5})S_2$. Refinement was a joint refinement using XRD (Supplementary Figure 2) and neutron (Supplementary Figure 3) powder data.

$LiV_{0.5}Ti_{0.5}S_2$: Space Group			P -3 m 1			
Lattice Parameters - **a**			3.4247(7)Å			
**c**			6.1582(2)Å			
Refinement parameters - R_{exp}			7.06%			
R_{wp}			6.32%			
R_p			6.16%			
Atom	X/a	Y/b	Z/c	Wyckoff symbol	Fractional Occupancy	B_{iso}
Li	0	0	0.5	1b	0.92(3)	1.8(2)
V/Ti	0	0	0	1a	0.51/0.49(2)	0.75(7)
S	1/3	2/3	0.2323(4)	2d	1	0.51(3)
